# Charge transfer between lead halide perovskite nanocrystals and single-walled carbon nanotubes†

**DOI:** 10.1039/c9na00766k

**Published:** 2020-01-02

**Authors:** Parul Bansal, Xiangtong Zhang, Hua Wang, Prasenjit Kar, William W. Yu

**Affiliations:** Department of Chemistry, Indian Institute of Technology Roorkee Uttarakhand 247667 India kar.prasen@gmail.com prkarfcy@iitr.ac.in; Department of Chemistry and Physics, Louisiana State University Shreveport Louisiana 71115 USA wyu6000@gmail.com

## Abstract

A charge transfer study between lead halide-based perovskite nanocrystals and single-walled carbon nanotubes (PNC@CNT nanocomposite) was performed. Solution-processed MAPbX_3_ PNCs displayed very bright luminescence, but it quenched in the presence of CNTs. This was attributed to the electron transfer from PNCs to CNTs. The detailed changes in fluorescence lifetime were investigated through time-correlated single-photon counting (TCSPC), which suggested mixed static and dynamic quenching along with a decrease in the lifetime. Morphological changes were investigated *via* transmission electron microscopy (TEM) and attributed to the incorporation of PNCs on long CNTs. Also, the PNC@CNT nanocomposite was explored for photoinduced current response, which indicated an ∼3 fold increase in photoconductivity under light illumination (with a 1 mV bias). This electron transfer study between PNCs and CNTs contributes to the exploration of charge dynamics.

## Abstract 2

A charge transfer study between lead halide-based perovskite nanocrystals and single-walled carbon nanotubes (PNC@CNT nanocomposite) was performed.

## Graphical abstract

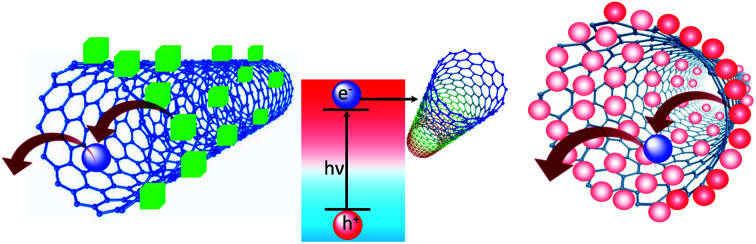

## 1. Introduction

Metal halide perovskites^1^ have emerged among the superior materials for optoelectronic devices^2–4^ including light-emitting diodes,^4^ solar cells,^5,6^ photodetectors, lasers, and in light harvesting.^2,3,8–12^ The efficacy of these applications relies on good absorption characteristics, easy charge carrier formation and transport in these materials.^2,13–15^ Besides, the low-cost solution synthesis of these perovskite nanocrystals (PNCs) results in intense fluorescence and nearly unity quantum yield.^2,16–21^ On the other hand, the strong confinement of carbon nanotubes (CNTs) to one dimension has also attracted the attention of the scientific community in the areas of charge transport, field emission, gas storage, photodetectors and other photovoltaic applications.^22,23^ Advancement in this research field has been probed through various photo-responsive CNT-based nanocomposites with organic fluorophores and inorganic moieties including conventional quantum dots (*e.g.*, CdSe/ZnS).^24–28^ The mechanistic investigation of CNTs and quantum dots revealed that the excited states of fluorescent quantum dots donate either excited electrons or holes to CNTs, which leads to the quenching of the photoluminescence.^25^

PNCs have size-dependent properties^7,17^ because of the quantum confinement effect, whereas CNTs have high electron transfer properties because of their nanoscale size, large surface area and direct path for charge transport.^29–31^ The formation of nanocomposites by combining the properties of both materials will lead to new avenues of opportunities in the field of nanotechnology. The photo-excited electron can be transferred from electron donor PNCs to electron acceptor CNTs. Earlier investigations on electron migration through static fluorescence quenching have been performed by Nair *et al.* using FAPbBr_3_ and fullerene C_60_.^32^ Kuang and coworkers studied the improvements in the charge transfer and light-harvesting properties of mixed cesium lead halide PNCs and CNTs.^33^ Chen and colleagues recently reported graphene sandwich stable PNC light-emissive ultrasensitive and ultrafast broadband phototransistors.^34^

**Fig. 1:**
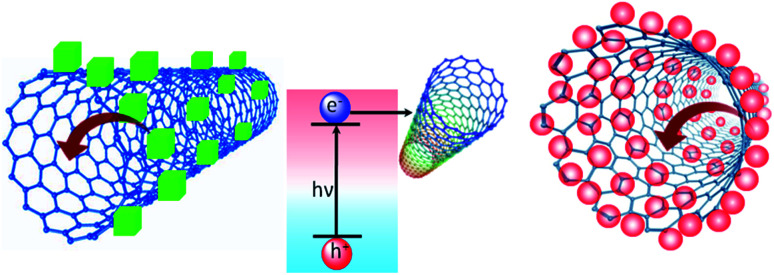
Schematic representation of electron transfer from MAPbX_3_ to a single-walled carbon nanotube.

Herein, we report a detailed electron transfer study using single-walled CNTs and methylammonium lead halide PNCs. The preference of single-walled CNTs over C_60_ fullerene is due to their superior electron transfer properties. Also, we have investigated the steady-state photoluminescence and the corresponding time-resolved lifetime, through which we have found a mixture of static and dynamic quenching (Fig. 1). The methods of preparation of MAPbBr_3_, MAPbI_3_ and CNT solutions are detailed in the ESI.†

**Fig. 2:**
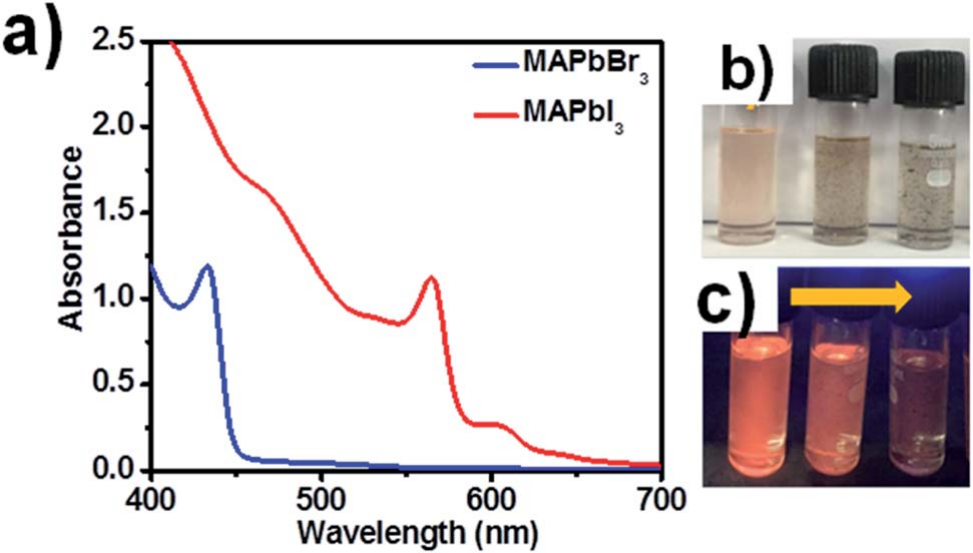
(a) Absorption spectra of MAPbBr_3_ and MAPbI_3_ PNCs in the presence of CNTs. (b) Daylight images of MAPbI_3_ solution with increasing concentration of CNTs (from left to right). (c) MAPbI_3_ solution with increasing concentration of CNTs (from left to right) under UV light.

The absorption spectra of blue luminescent MAPbBr_3_ and red luminescent MAPbI_3_ are shown in Fig. 2a, and images of MAPbI_3_ PNCs in daylight and UV light are presented in Fig. 2b and c. Images of green and blue luminescent MAPbBr_3_ PNCs in the presence of increasing concentrations of CNT are shown in Fig. S1.† XRD patterns of synthesized perovskite nanocrystals are shown in Fig. S2† for blue luminescent MAPbBr_3_ and red luminescent MAPbI_3_, confirming the presence of the cubic and tetragonal phases in MAPbBr_3_ and MAPbI_3_ PNC.^17^ Absorption peaks of MAPbBr_3_ solution were observed at 462 nm and at 563 and 604 nm for MAPbI_3_ in the presence of CNTs. MAPbX_3_ PNCs are highly fluorescent and their optical properties can be easily modified by size variation. The addition of CNTs to PNC solution leads to the adhering of quantum dots to the walls of CNTs because of van der Waals interactions. In our case, we sonicated the CNT solution for a longer time to separate the CNT bundles and increase the surface area of CNTs so that electron transfer would take place more efficiently. Pristine CNTs (not functionalized) were used, and the presence of oleic acid and oleylamine helped the adhesion of PNCs to the walls of the CNTs. To monitor the effect of solvent (*i.e.* CHCl_3_), we performed the emission studies in the presence of CHCl_3_ (without the addition of CNT) and with respect to time as shown in Fig. S3,† indicating the negligible effect of the solvent, which confirmed that the quenching is due to the adhesion of perovskite nanocrystals to CNTs.

**Fig. 3:**
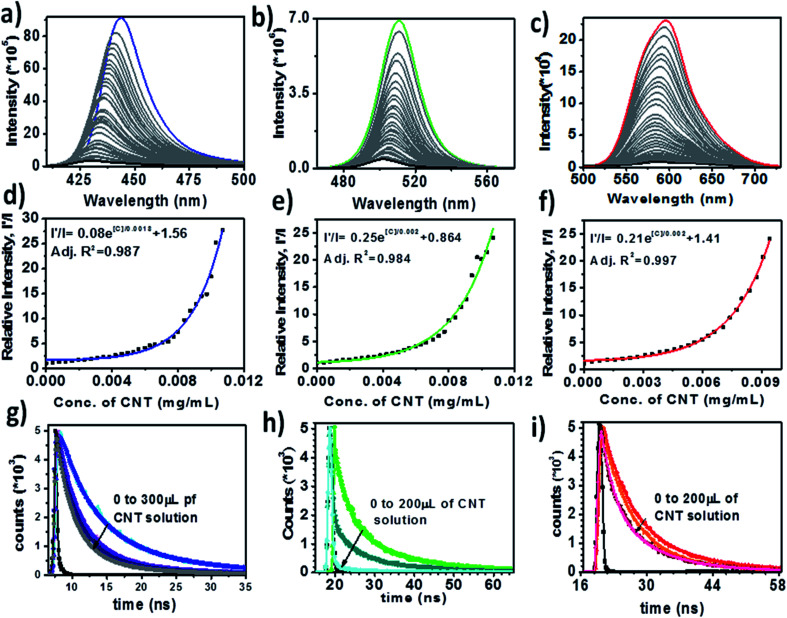
Photoluminescence (PL) quenching of (a) blue luminescent MAPbBr_3_, (b) green luminescent MAPbBr_3_, and (c) red luminescent MAPbI_3_ PNCs with CNTs. (d) Relative PL intensity changes with CNT concentration on the quenching of (d) blue, (e) green, and (f) red luminescent PNCs. TCSPC measurements of (g) blue, (h) green and (i) red luminescent PNCs on the addition of CNTs. The CNTs were added in the form of 0.01% w/v solution.

As shown in Fig. 3a–c, there was almost 100% fluorescence quenching of blue, green and red luminescent PNCs with CNTs, which is clear evidence that the electron transfer takes place from the electron donor PNCs to the acceptor CNT bundles. The quenching experiment was performed with the sequential addition of CNT solution (0.01% w/v in chloroform) from 10 μL to 300 μL. We observed blue shifts in all PNC peaks as compared to previous reports^17^ due to the dilution of samples before quenching studies. In Fig. 3a–c, the emission peaks of the blue luminescent MAPbBr_3_, green luminescent MAPbBr_3_ and red luminescent MAPbI_3_ (without CNTs) were observed at 443 nm, 510 nm and 596 nm, respectively.

We observed almost 100% quenching with the addition of 300 μL of CNT solution for all three PNCs. Apart from the quenching of the emission peaks, we also observed blue shifts in the spectra (initially) on increasing the amount of CNT (predominantly for blue luminescent MAPbBr_3_ PNCs). The rate of PL quenching was nonlinear as shown in Fig. 3d–f. A possible reason for these PNC@CNT nanocomposites to give nonlinear Stern–Volmer plots is the presence of both static and dynamic quenching.^35^Fig. 3d–f shows curves along with exponential equations and rate constants for blue, green and red luminescent PNCs, respectively.

The most efficient way to determine whether quenching is static, dynamic or both is through PL decay lifetime measurements. If the lifetime values remain unchanged in the presence of CNTs, it might be due to static quenching, as previously reported with FAPbBr_3_ and fullerene.^28^ However, in the case of MAPbX_3_ PNCs there was a decrease in the lifetime of PNCs with increasing the amount of CNTs, which was attributed to the presence of static and dynamic quenching as shown in Fig. 3g–i. The average lifetimes along with the details of parameters for blue, red and green luminescent PNCs in the presence of CNT solution are listed in Table 1. The average lifetime was calculated by the following formula:*τ*_avg_ = (*A*_1_*τ*_1_^2^ + *A*_2_*τ*_2_^2^ + *A*_3_*τ*_3_^2^)/(*A*_1_*τ*_1_ + *A*_2_*τ*_2_ + *A*_3_*τ*_3_)where *A*_1_, *A*_2_ and *A*_3_ are weighing parameters, *τ*_1_, *τ*_2_ and *τ*_3_ are the corresponding lifetimes and *τ*_avg_ is the average lifetime.

**Table 1:** TCSPC details of PNC solutions in the presence of CNT

Sample	CNT volume (μL)	*A*_1_	*A*_2_	*A*_3_	*τ*_1_ (ns)	*τ*_2_ (ns)	*τ*_3_ (ns)	*τ*_avg_ (ns)
Blue MAPbBr_3_	0	32.24	53.35	14.40	4.23	10.53	60.08	37.40
	100	48.31	41.89	9.80	2.82	8.14	53.82	31.40
	200	34.87	9.84	55.29	8.93	55.41	2.88	32.92
	300	37.35	9.44	53.22	7.69	46.58	2.45	26.88
Green MAPbBr_3_	0	41.95	27.51	30.54	9.05	0.12	32.63	26
	100	3.76	3.96	92.27	6.12	24.96	0.044	20
	200	9.04	12.93	78.03	2.75	17.81	0.25	15.19
Red MAPbI_3_	0	37.60	54.26	8.14	5.63	12.03	52.30	24.26
	100	47.62	47.56	4.86	5.41	10.26	49.35	18.51
	200	22.10	67.51	10.39	2.56	9.03	31.68	16

**Fig. 4:**
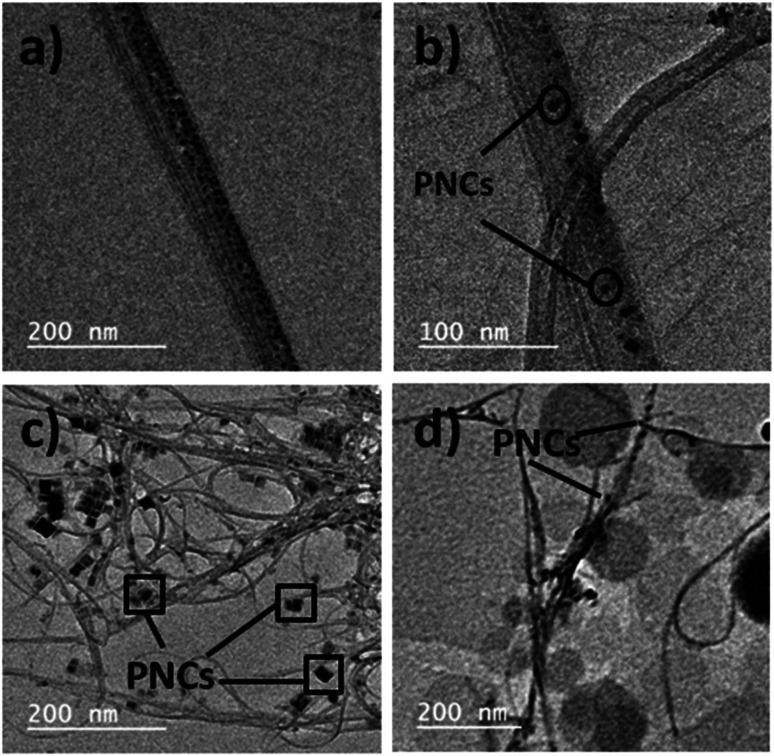
TEM images of (a), (b) blue, (c) green and (d) red luminescent PNCs in the presence of CNTs.

The X-ray diffraction (XRD) pattern of MAPbBr_3_@CNT is provided in Fig. S4,† showing the presence of perovskite peaks. TEM images of blue, green and red luminescent MAPbX_3_ PNCs are given in Fig. S5.† From Fig. S5,† the tunable morphology is directly evidenced by the TEM images, where the morphology changes from quantum dots to nanoplates to quantum dots by shifting from blue-green-red luminescent MAPbX_3_ PNCs, which leads to adhesion to CNTs as shown in Fig. 4. These interactions are due to the electron migration or transfer from electron donor halide perovskites to electron acceptor CNTs. TEM images of PNC@CNT nanocomposites provide direct evidence of PNCs adhering to CNTs as shown in Fig. 4. The typical Fourier transform-infrared spectroscopy (FT-IR) vibration modes are presented in Fig. S6.† XPS analysis of green luminescent MAPbBr_3_ in the presence of CNT confirms the presence of the elemental composition of PNC with the main peaks of Pb 4f, C 1s, N 1s and Br 3d in narrow scans as shown in Fig. S7.†

## 2. Photoresponse study

**Fig. 5:**
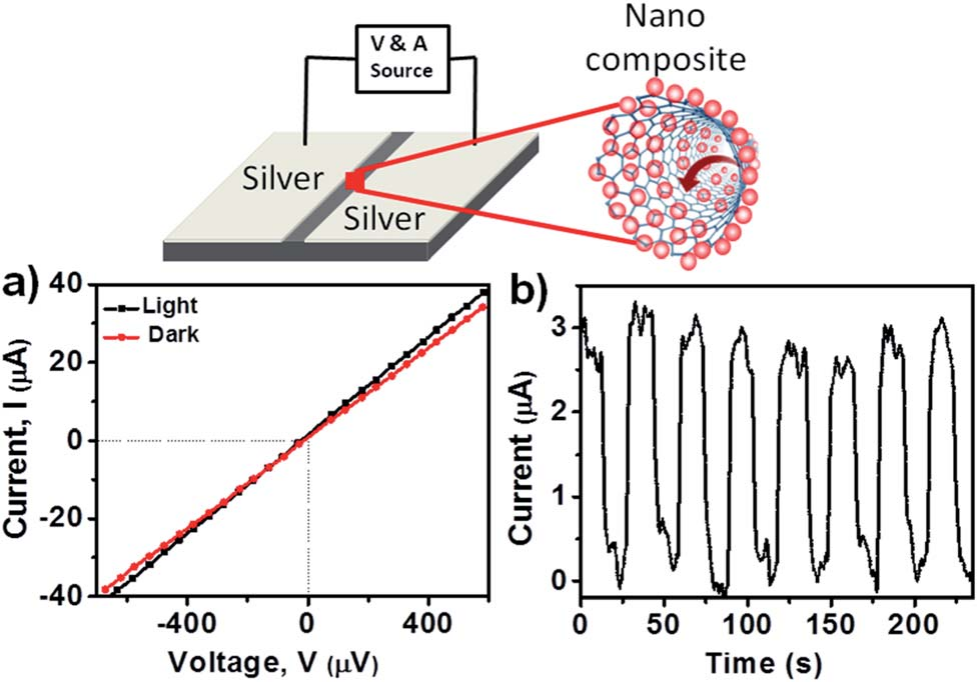
Schematic illustration of the fabricated device (top); (a) the *I*–*V* curve for MAPbI_3_@CNT in the presence and absence of light and (b) the corresponding *I*–*t* curve for MAPbI_3_@CNT.

A prototype device was fabricated to investigate the conductive properties of the PNC@CNT nanocomposite. A schematic illustration is provided in Fig. 5 for measuring the photoconductivity. The device was made by two silver electrodes separated by 0.5 mm on a silica substrate and drop-casting the concentrated MAPbI_3_@CNT nanocomposite sample to connect the two electrodes. The photoinduced current was measured on a Keithley 2612B source meter while irradiating the sample with a daylight lamp having a brightness of 1200 lumens. Fig. 5a shows the current–voltage curve of the nanocomposite in the dark and in the presence of light, displaying excellent ohmic responses. The resistance in the presence of light was 14.2 Ω, whereas in the dark it was 15.37 Ω, which confirms that irradiation in light leads to an increase in current (Fig. 5a). Photoinduced charge was generated in this device system upon irradiation with light.^21^ It was found that the photoconductivity increased ∼3 fold under light illumination at 1 mV voltage (Fig. 5b) and the photocurrent was basically constant over time as shown in Fig. 5b, indicating the good stability of the nanocomposite over a number of cycles. It has been confirmed that the presence of CNT alone does not lead to the change in current under light irradiation.^36,37^ As such, the increase in the current (Fig. 5a and b) was due to the presence of PNCs and that led to the charge transfer between CNTs and PNCs and completed the circuit. For multiple current cycle investigations, the light was turned on and off with a 15 s interval.

Photocurrent (*I*_ph_) was evaluated by *I*_ph_ = |*I*_l_ − *I*_d_| where *I*_d_ is the current in the dark and *I*_l_ is the current under illumination. As shown in Fig. 5b, the PNC@CNT nanocomposite exhibited a photocurrent which led to a difference in current under light irradiation. The device exhibited a photocurrent of 2.92 μA because of the adhesion of PNCs on CNTs through van der Waals interactions. Applied bias led to the generation of charge carriers that migrated from the electron donor PNCs to electron acceptor CNTs, leading to the enhancement of the current. These results indicate that charge transfer between PNCs and CNTs produced a noticeable photocurrent, which paves the way towards new optoelectronic and nanosensor-based applications.

## 3. Conclusions

In summary, we studied the charge transfer behaviour between lead halide based perovskites and carbon nanotubes. Fluorescence quenching and lifetime studies revealed the presence of static as well as dynamic quenching. TEM morphological studies and PL quenching also indicated significant interaction enhancement between PNCs and CNTs. Photoconductive perovskite nanocomposites have opened new avenues for solar cells, sensor-based nanodevices and light-driven optoelectronics.

## Conflicts of interest

There are no conflicts of interest to declare.

## Supplementary Material

NA-002-C9NA00766K-s001
