# Supplementary material for: Charge transfer between lead halide perovskite nanocrystals and single-walled carbon nanotubes
Source: Nanoscale Adv. 2020 Jan 2;2(2):808–13. doi: 10.1039/c9na00766k (PMC9417507; doi:10.1039/c9na00766k)
Supplement: NA-002-C9NA00766K-s001 [file NA-002-C9NA00766K-s001.pdf]

Electronic Supplementary Material (ESI) for Nanoscale Advances.
This journal is © The Royal Society of Chemistry 2020

Supporting Information

Charge Transfer between Lead Halide Perovskite Nanocrystals and Single Walled Carbon Nanotubes

Parul Bansal[a,b], Xiangtong Zhang[b], Hua Wang[b], Prasenjit Kar[a,*], William W. Yu[b,*]

[a] Department of Chemistry, Indian Institute of Technology Roorkee, Uttarakhand 247667, India

kar.prasen@gmail.com, prkarfcy@iitr.ac.in

[b] Department of Chemistry and Physics, Louisiana State University Shreveport, Louisiana 71115, United States

wyu6000@gmail.com

Instrumentation Details

UV-Vis absorption spectroscopy and photoluminescence spectroscopy Shimadzu UV-Vis 2450 spectrophotometer was used for recording UV-Vis absorption spectra in the range of 200-650 nm. Photoluminescence spectra were taken by Horiba scientific Fluoromax-4C spectrophotometer. A quartz cuvette of 10 mm path length and 3 ml volume was used for collecting the spectra.

Powder XRD was carried out on Bruker -D8 Advance having Target Cu and accelerating voltage 40kV from 10˚ to 50˚. Thin film samples were prepared on silica glass.

TCSPC studies Fluorescence lifetime decay measurements were recorded on 1 cm quartz cell on a Horiba Jobin Yvon Fluorocube Fluorescence Lifetime System equipped with NanoLEDs and LDs as the excitation source and an automated polarization accessory (Model 5000 U-02).

Fourier Transform Infrared Spectroscopy (FTIR) Infrared spectra (IR) of materials were recorded by using Thermo Scientific Nicolet 6700.

Transmission Electron Microscopy (TEM) TEM study was carried out by FEI TECHNAI G^2 20 S-TWIN. A drop (5-10μL) of diluted samples was placed on carbon coated copper grid. Again a drop was added before drying it. Afterwards drying was carried at ambient temperature.

X-Ray Photoelectron Spectroscopy (XPS) Thin film of perovskites has been studied on XPS with model no. PHI 5000 VersaProbe III for surface analysis.

Keithley Sourcemeter with Model 2612B and software Kickstart 2.1.0 version was used for photoresponse studies. Light source was a daylight LED (12 W) used for current-time studies.

Experimental

Chemicals and Materials

All the chemicals and solvents were purchased from commercial sources and used as received. Lead (II) bromide (99%), lead (II) iodide, methylamine solution (33 wt% in absolute ethanol) and oleylamine, carbon nanotube were purchased from Sigma-Aldrich. Other reagents including chloroform (Rankem), ethanol, N,N-dimethylformamide (Thomas Baker), oleic acid, hydroiodic acid and hydrobromic acid (48 wt% in water, SRL) were purchased from local suppliers. Molecular sieves were used to remove trace water of the solvents.

Methylammonium Iodide/Bromide (MAI/MABr) Synthesis. Briefly, in a 50 ml flask, 5.14 ml CH_3NH_2, 33 wt% in absolute ethanol, was mixed with 10 ml of ethanol. Then, at room temperature, 6 ml 57% water solution of the HI/HBr was added dropwise with continuous stirring. The obtained solution was placed in a rotary evaporator at 60°C for removing all solvents. Then after several times of washing with diethyl ether, CH_3NH_3I was dissolved in ethanol and precipitated with diethyl ether twice. The product was recrystallized once.

$CH_3NH_3PbI_3$ Nanocrystals (Red Luminescence)

0.1 mmol of CH_3NH_3I and 0.1 mmol of PbI_2 were dissolved in 1 ml of DMF forming 0.1 mM solution. 100 μL of oleic acid and 200 μL of oleylamine were then added simultaneously. Next 100 μL of this mixture was injected into 3 ml of anti-solvent chloroform media. The obtained solution was observed under UV chamber showing red color.

$CH_3NH_3PbBr_3$ Nanocrystals (Blue Luminescence)

0.1 mmol of CH_3NH_3Br and 0.1 mmol of $PbBr_2$ were dissolved in 1ml of DMF forming 0.1mM solution. 100 μL of oleic acid and 200 μL of oleylamine has been added simultaneously. Next 100 μL of this mixture was injected into 3ml of anti-solvent chloroform media. Obtained solution was observed under UV chamber was showing blue color.

$CH_3NH_3PbBr_3$ Nanocrystals (Green Luminescence)

0.1 mmol of CH_3NH_3Br and 0.1 mmol of $PbBr_2$ were dissolved in 1ml of DMF forming 0.1 mM solution. 200 μL of oleic acid and 38 μL of oleylamine were added simultaneously. Next 100 μL of this mixture was injected into 3 ml of anti-solvent chloroform media. The obtained solution was observed under UV chamber showing green luminescence.

All of the solutions were diluted with 50 ml of solvent (chloroform) before studies of quenching in emission.

CNT Solution

0.01% w/v CNT solution is prepared and sonicated for 25 minutes before using it in formation of nanocomposite.

$MAPbI_3$@CNT Sample Preparation for Photoresponse Study

Prepared $MAPbI_3$ PNCs were centrifuged and re-dispersed in chloroform to get concentrated solution. 1 ml of $MAPbI_3$ solution was mixed with 500 µL of CNT solution and sonicated for 20 minutes. Later, solution was drop casted by scratching between silver electrodes (using blades) to measure photoresponse studies.

Preparation of Silver Electrodes and Drop Casting the Nanocomposites

1. Got 1 g of silver nitrate and 1 g of sodium hydroxide in separate containers and added enough water to both to dissolve them.
2. Mixed two solutions together and add ammonia to dissolve the precipitates
3. Added 4 g of sugar to dissolve them
4. Placed the silica glass in the glass plate and put it on hot flame (medium temperature) and heat it for 2 minutes.
5. Solution turned to cream colour and put glass out of plate and cooled it.
6. The silica substrate was coated with silver. Kept it overnight to get it dry.
7. The solution of nanocomposite was sonicated for 25 minutes before drop casting it in between the silver electrodes. The solvent was evaporated in ambient conditions.

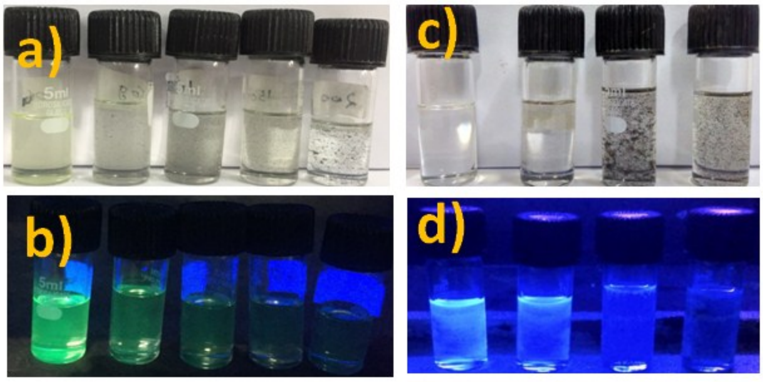

Figure S1 a) Daylight image and b) image under UV light of green luminescent $MAPbBr_3$ PNCs in the presence of increasing concentration of CNT c) Daylight image and d) image under UV light of blue luminescent $MAPbBr_3$ PNCs in the presence of increasing concentration of CNT.

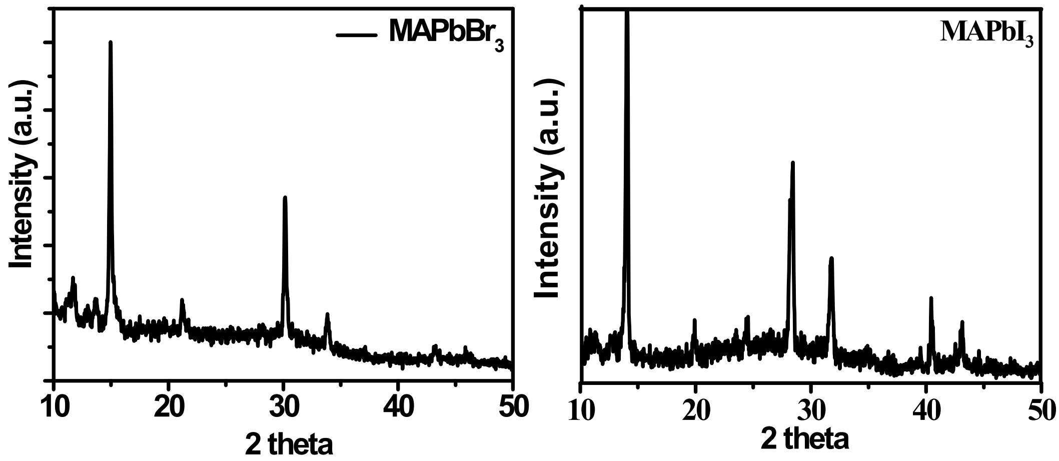

Figure S2 XRD patterns for $MAPbBr_3$ and $MAPbI_3$ perovskite nanocrystals.

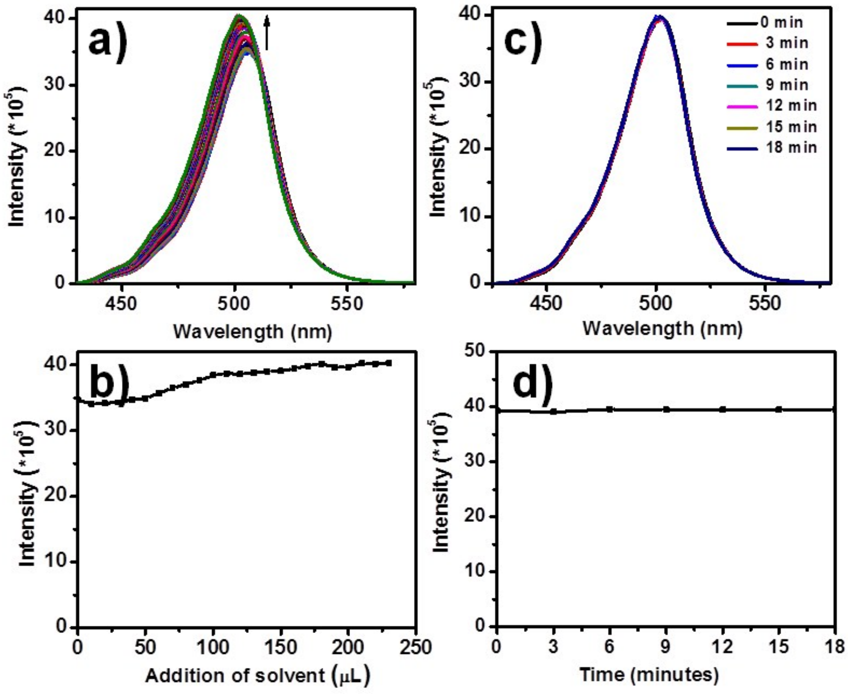

Figure S3 a) Emission spectra of $MAPbBr_3$ perovskite nanocrystals till addition of 250 μL of solvent b) Intensity vs addition of solvent for green $MAPbBr_3$ Perovskite nanocrystals c) time

dependent emission spectra of $MAPbBr_3$ perovskite nanocrystals d) Intensity vs time spectra of $MAPbBr_3$ perovskite nanocrystals

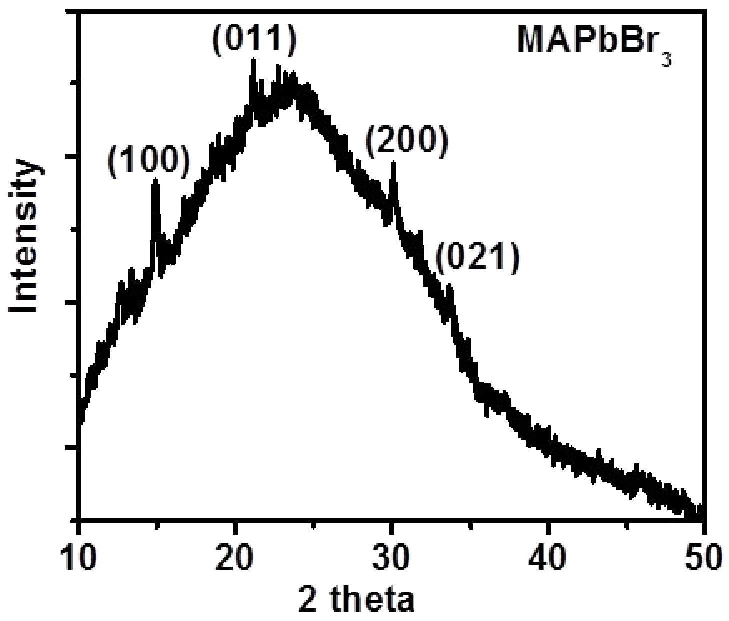

Figure S4 XRD of green luminescent $MAPbBr_3$ in the presence of CNT.

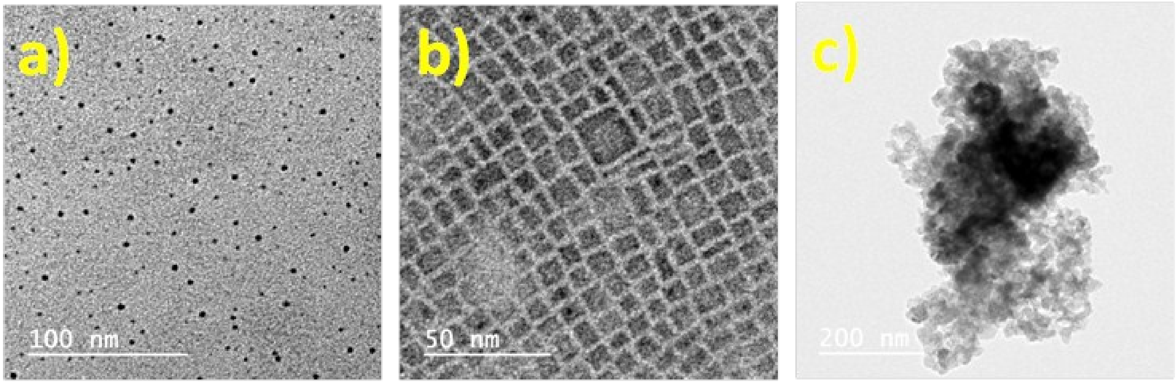

Figure S5 a) TEM images of blue luminescent $MAPbBr_3$ PNCs b) TEM images of green luminescent $MAPbBr_3$ PNCs c) TEM images of red luminescent $MAPbBr_3$ PNCs.

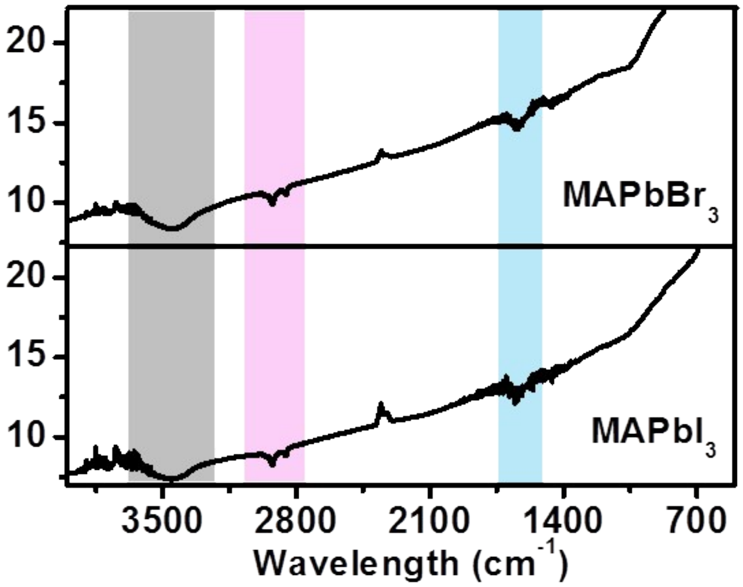

Figure S6 FT-IR spectroscopic details of PNCs in the presence of CNT.

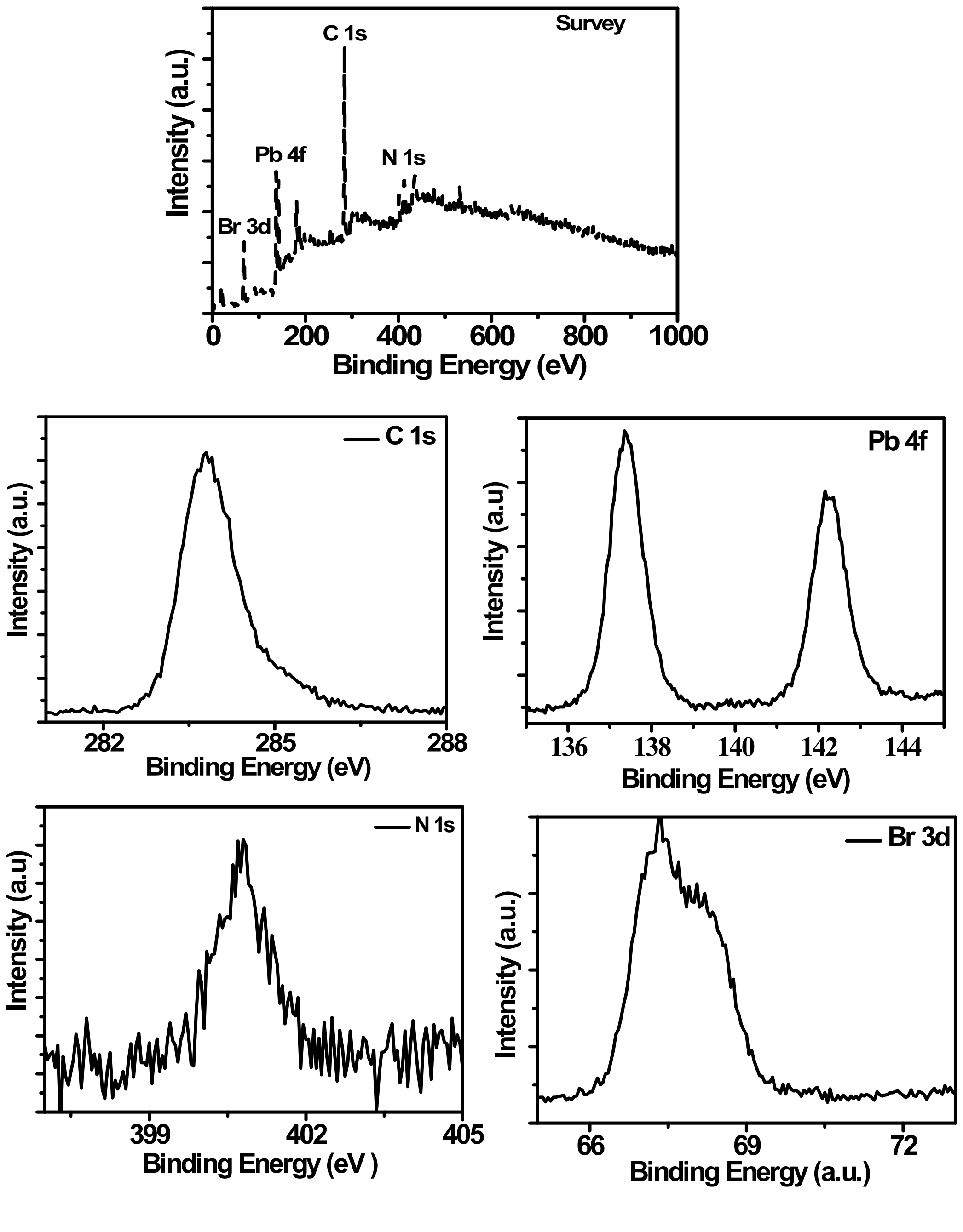

Figure S7 Survey and narrow scans through XPS for elemental analysis.
